# Different radius of curvature at the talus trochlea from northern Chinese population measured using 3D model

**DOI:** 10.1186/s13018-024-04751-7

**Published:** 2024-04-27

**Authors:** Shixun Wu, Shizhang Liu, Minggang Huang, Zhe Liu, Jiyuan Shi, Ming Ling

**Affiliations:** 1 Department of Orthopedics Surgery, Shaanxi Provincial People’s Hospital, No. 256 Youyi West Road, Xi’an, Shaanxi 710068 China; 2 Department of CT, Shaanxi Provincial People’s Hospital, Xi’an, Shaanxi 710068 China; 3 Key Laboratory of Bone Joint Disease Basic and Clinical Translation of Shaanxi Province, Xi’an, Shaanxi 710068 China

**Keywords:** Curvature of talar trochlea, Talus necrosis, Osteoarthritis, Artificial ankle prosthesis, Computer-aided design

## Abstract

**Background:**

To analyze the curvature characteristics of the talus trochlea in people from northern China in different sex and age groups.

**Methods:**

Computed tomography scanning data of talus from 61 specimens were collected and constructed as a three-dimensional model by Materialise’s Interactive Medical Image Control System(MIMICS) software, anteromedial(AM), posteromedial(PM), anterolateral(AL), and posterolateral(PL) edge, anterior edge of medial trochlea, posterior edge of medial trochlea and anterior edge of lateral trochlea were defined according to the anatomical landmarks on trochlear surface. The curvature radii for different areas were measured using the fitting radius and measure module.

**Results:**

There were significant differences among the talus curvatures in the six areas (F = 54.905, *P* = 0.000), and more trends in the analytical results were as follows: PM > PL > MP > AL > MA > AM. The average PL radius from specimens aged > 38 years old was larger than that from specimens aged < = 38 years (t=-2.303, *P* = 0.038). The talus curvature of the AM for males was significantly larger than that for females (t = 4.25, *P* = 0.000), and the curvature of the AL for males was larger than that for females (t = 2.629, *P* = 0.010). For observers aged < = 38 years, the AM curvature of the right talus in the male group was significantly larger than that in the female group (*P* < 0.01). In age < = 38years group, the MA curvature of right talus in male was significantly larger than in female group(*P* < 0.01), fitting radius of talus for male (21.90 ± 1.97 mm) was significantly greater than female of this(19.57 ± 1.26 mm)(t = 6.894, *P* = 000). The average radius of the talus in the male population was larger than that in the female population.

**Conclusion:**

There was no significant relationship between age and talus curvature for males and females. The radius of curvature in the posterior area was significantly larger than that in the anterior area. We recommend that this characteristic of the talus trochlea should be considered when designing the talus component in total ankle replacement (TAR).

**Supplementary Information:**

The online version contains supplementary material available at 10.1186/s13018-024-04751-7.

## Background

With the continuous improvement of people’s understanding of ankle anatomical structure and disease diagnosis and treatment, ankle replacement technology and prosthesis design are constantly updated. TAR was considered reliable ultimate treatment planning at the end stage of ankle osteoarthritis, talus necrosis, and Kashin-Beck disease(KBD).

Finite element analysis (FEA) of the ankle joint shows significant instability when the area of the defect exceeds 6 mm * 6 mm [1]. Osteochondral autologous transplantation (OAT) technology has been used for treating osteochondral lesions of the talus [2–4]; however, the effectiveness of treatment still needs to be confirmed by clinical follow-up results over the long term. Recently, custom 3D-printed titanium implants for treating critically sized bone defects did not need subsequent surgery (1-year follow-up) [2]. This result suggests that 3D-printed titanium alloy implants can replace damaged bone.

Indications for total talus replacement (TTR) have expanded in recent years, and encouraging efficiency has been found, such as following traumatic injury [5], tumor [6, 7], arthrodesis, and idiopathic avascular necrosis [8, 9]. However, TTR is not an indication for total ankle arthritis, and adjacent joint arthritis was observed as the most common complication after TTR [10].

The biological models during FEA of ankle hemiarthroplasty had the largest contact areas and smallest peak contact pressures [11]. Patients with high BMI ( > = 25 kg/m2) need longer treatment procedures for osteochondral lesions of the talus and have poorer 36-item short-form health survey scores at 24 months post-operation [12]. Because 60% of the surface of the talar dome is covered by articular cartilage [13], the blood supply is very poor. These factors lead to easier talar disease, such as arthritis and talar osteonecrosis.

The Scandinavian TAR system is the only fourth-generation ankle prosthesis used in the USA, but the design of the talar component is symmetrically cylindrical [14], which is not similar to the true talus trochlear shape with different radii curvatures. The design of fourth-generation ankle replacement should consider many factors, including decreased bone resection, minimized disruption of the anterior tibial cortex, anatomically contoured distal tibial trays, and talar components with different curvatures of radii [14].

Due to various complications [15], the design of ankle prostheses is not ideal. The first- and second-generation designs were gradually abandoned in the medical market. At present, there are still ankle prostheses with excellent performance, but the ankle talus components are not similar to the normal talus shape design, which may lead to various clinical symptoms after TAR and the reasons for the failure of replacement. Therefore, we use 3D model technology to study the shape of the talus in a realistic way. It will provide believable evidence for optimizing the design of talar prostheses.

## Materials and methods

### Specimen acquisition and computer-assisted tool

A SOMATOM Definition Flash dual-source CT machine (Siemens Healthineers, Forchheim, Germany) was selected to scan the ankle joint of subjects from Shaanxi Province People’s Hospital. Scan parameters: 120 kV, 205.50 mAs, layer thickness: 0.5–1 mm, all DICOM images (521 px×512 px) in 336 layers for each subject, were imported into a standard segmentation software - MIMICS 17.0 software (Materialise, Leuven, Belgium), the region of interest (ROI) were extracted using both “Thresholding” and “region growing” module. All 3D models of the talus were automatically produced by the “calulate 3D from mask” functional block and then imported into Geomagic Stuido 12 to refine the model structure and improve the accuracy of the measurement results. Finally, the STL for the talar model was imported into 3 matic software for anatomical measurement. The computer workstation included a Lenovo thinkpad, Windows 7–64 bit operating system, Intel (R) Core(TM) i7-4600 processor, 8 GB of running memory, and 256 SSD hard disk. All the subjects signed the participant consent form. This research was approved by the Ethics Committee of Shaanxi Provincial People’s Hospital (No. SPPH-LLBG-17-3.2).

The CT data of the talus from subjects were collected in both outpatient and inpatient departments of Shaanxi Provincial People’s Hospital. The inclusion criteria were as follows: patients without/with a history of trauma but without fracture or dislocation of the talus. The exclusion criteria were as follows: (1) talar fracture; (2) congenital or acquired skeletal deformity; (3) necrosis of the talus caused by KBD/Rheumatoid arthritis; and (4) talar tumor from various pathological types.

### Calibration of the coordinate system

To ensure that each talus model had the same three-dimensional position in the 3matic 11.0 software, the object coordinate system (OCS) was formatted to the world coordinate system (WCS) in the 3matic 11.0 software. Origin 0 (0,0,0) was defined as the centerpoint in the WCS and is an intersection point caused by 3 orthogonal planes. The XY plane (axial plane), YZ plane (sagittal plane), and ZX plane (coronal plane) were generated separately.

### Definition of anatomical landmarks on the surface from the talus trochlea

**Fig. 1:**
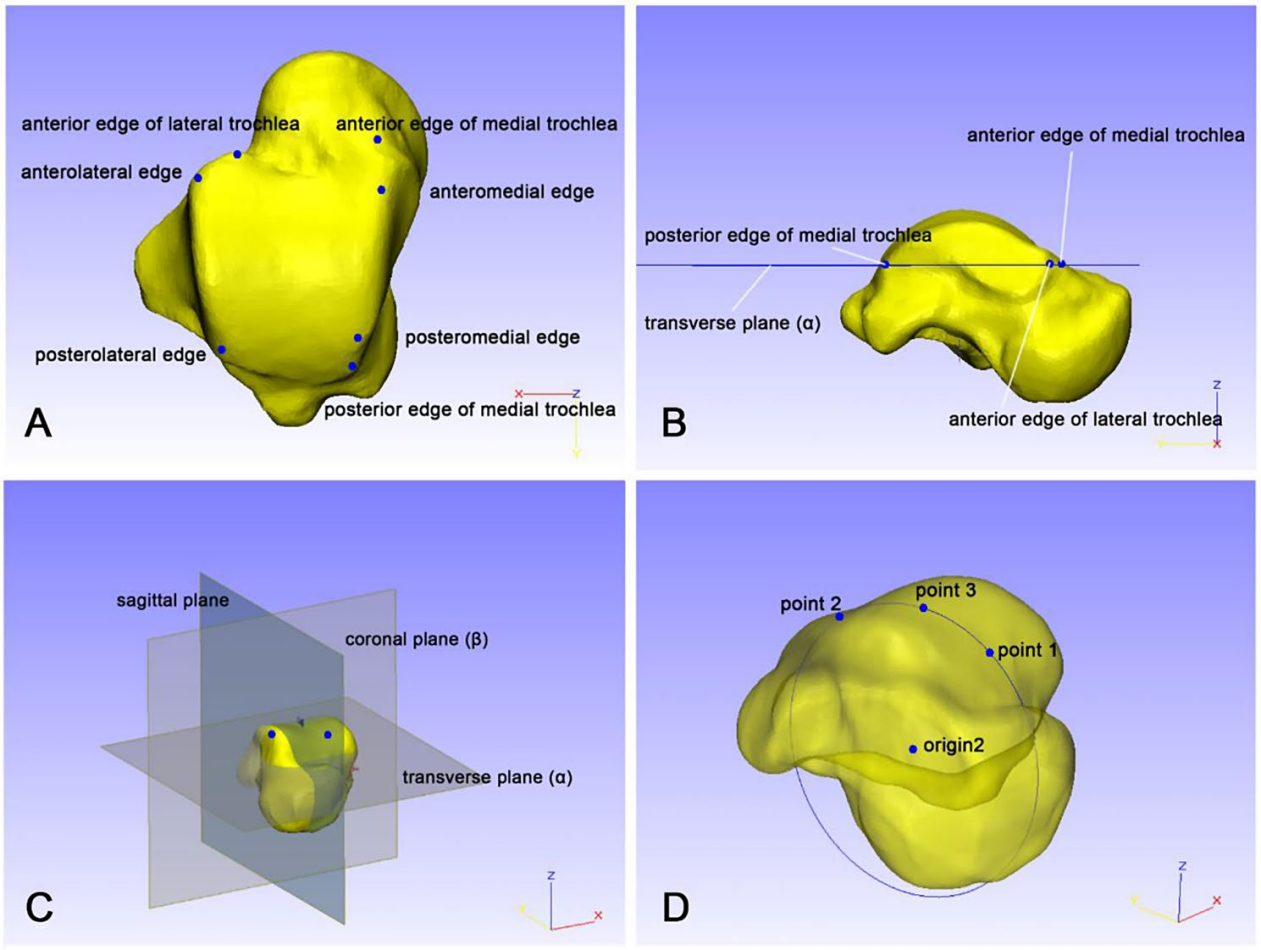
Standardization of the talus model based on anatomical landmarks. (**A**. Seven anatomical top points on the talus trochlea; **B**. Determining the transverse plane α based on three anatomical points; **C**. Determining the sagittal plane and coronal plane based on the transverse plane α; **D**. Fitting the origin point of the talus by three points)

According to a previously published method [16], the AM, PM, AL, and PL edges of the talar trochlea were defined, as shown in Fig. 1A. The anterior edge of the medial trochlea and anterior edge of the lateral trochlea were defined as the most anterior point of medial border and lateral border in trochlea, respectively. The posterior edge of the medial trochlea was defined as the most posterior point of the medial trochlea.

The transverse plane (α) was a datum plane created when passing through the anterior edges of the medial trochlea, posterior edges of the medial trochlea and anterior edge of the lateral trochlea (Fig. 1B). The coronal plane (β) was created as a datum plane perpendicular to α and passing through midpoint 1 (the anterior and posterior edges of the medial trochlea) and midpoint 2 (the anterior edge of the lateral trochlea and PL edge of the talar trochlea). The sagittal plane was set perpendicular to both the transverse plane (α) and coronal plane (β). (See Fig. 1C)

### Centerpoint of talus

The intersection (origin 1) of the three coordinate planes (transverse plane, sagittal plane and coronal plane) was calculated by 3matic; then, the midpoint (point 1) of the AM and AL edges and the midpoint (point 2) of the PM and PL edges of the trochlea were defined; next, the midpoint of point 1 and point 2 was marked and projected onto the trochlea surface (point 3). A circle was created according to point 1, point 2 and point 3, and the center of the circle (origin 2) was set as the center of the talus (see Fig. 1D).

Origin 1 and origin 2 coincided with origin 0, and the centerpoint of the talus coincided with the origin of the WCS. The X axis is created through origin 0 and perpendicular to the sagittal plane, the Y axis is perpendicular to the coronal plane (β) through origin 0, and the Z axis is through origin 0 perpendicular to the transverse plane (α). All parts and analytical primitives in work area were rotated make sure X axis perpendicular to ZY plane, Y axis perpendicular to ZX plane, Z axis perpendicular to YX plane. The coordinate system of the talus is coincident with the WCS.

### Top point on the trochlear surface

**Fig. 2:**
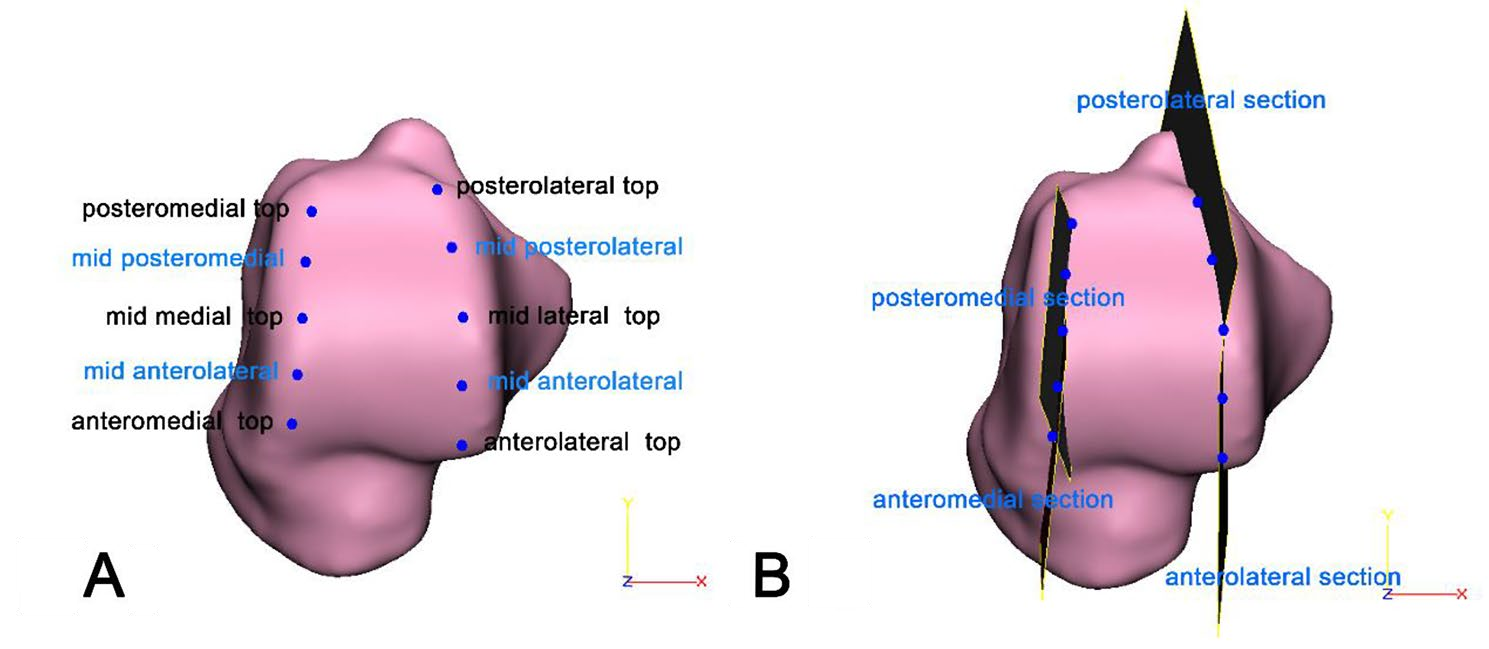
Definition of the top point in six areas (**A**) and four sections (**B**)

Six near coronal sections parallel to the ZY plane were produced, which passed through the AM edge, midpoint of the AM and PM edges, PM edges, AL edge, midpoint of the AL and PL edges, and PL edges previously determined [17]. The intersection top points between the trochlear surface and six near coronal sections were identified as the AM top, mid-medial top, PM top, AL top, mid-lateral top, and PL top. (Fig. 2A)

### Five nearly sagittal sections

According to the principle of using three points to determine a plane, an AM section was established when it contained the AM top, mid-medial top, and midpoint between these two tops; in a similar way, a PM section, AL section, and PL section were defined. Each radius of curvature was calculated using the *measure* module in 3matic( Fig. 2B). Additionally, the midsagittal section is the sagittal section in the WCS in 3matic 11.0 software.

### Measured radius of talus curvature

**Fig. 3:**
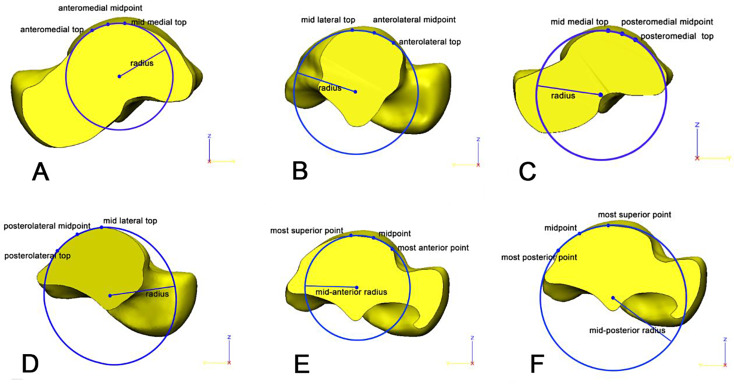
Curvature of talus in different areas(**A**,AM; **B**,AL; **C**,PM; **D**, PL; **E**,MA; **F**,MP.)

First, the talus was cut by the AM section, the midpoint between the AM top and midmedial top was defined based on the length (AM midpoint), and then a circle was established through the three points. The radius was recorded as the anteromedial curvature of the talus (Fig. 3A). In the same way, the talus was cut by the AL section, the midpoint between the AL top and midlateral top was defined based on the length (AL midpoint), and the AL curvature of the talus was defined as the radius of the circle established through three points (Fig. 3B).

Second, the talus was cut by the PM section, the midpoint between the PM top and midmedial top was defined based on the length (PM midpoint), and the PM curvature of the talus was the radius of the circle established through three points (Fig. 3C). The talus was cut by the PL section, the midpoint between the PL top and midlataral top was defined based on the length (PL midpoint), and the PL curvature of the talus was the radius of the circle established through three points (Fig. 3D).

Third, the talus was cut by the midsagittal section, the anterior point/superior point/posterior point was marked, the anterior midpoint and posterior midpoint were calculated and marked, and the mid posterior curvature and mid anterior curvature were established through a circle containing three points (Fig. 3E and F).

### Fitted radius of talus curvature based on the cylinder

**Fig. 4:**
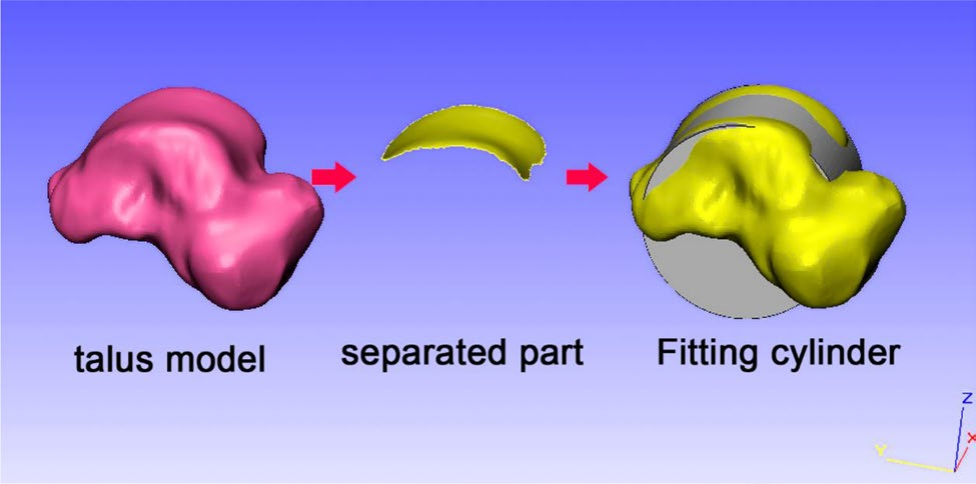
Schematic diagram for fitting the radius of curvature

Following the normalized coordinate system of the talus, the trochlear surface of the talus was separated as a new part, and an analytical cylinder was fitted using the separated trochlea of the talus. The radius of the analytical cylinder shown on the properties page is similar to the talus curvature (Fig. 4).

### Statistical analysis

All measurements of the 3D model in this study, including each trochlea of the talus, were measured by the same researcher. All data were collected and entered into Microsoft Excel 2016, SPSS17.0 statistical software package (SPSS Statistics for Windows, Version 17.0. Chicago: SPSS Inc.) was employed to identify significant differences. Independent sample t tests were used to identify the difference between two sets of data, paired *t* -tests were used to analyze the difference between the left and right trochlea of the talus, and the results are expressed as the mean ± standard deviation (‾x ± SD). The difference among multiple samples was tested by the LSD multiple comparison method; the relationship between the two variables was analyzed by bivariate correlation analysis. All statistical tests were two-sided, and *P* < 0.05 was considered statistically significant.

## Results

### Baseline information of collected data

All specimens from the CT dataset collected according to the inclusion criteria included 61 specimens (28 male and 33 female) that were reconstructed as 91 talus models, including 41 right ankles and 50 left ankles, 31 unilateral ankle joints and 30 bilateral ankle joints. The average age was 37.43 (14 y-69 y), and the median age was 38 years old.

### Curvature of talar radii

91 ankle joints were selected during the statistical analysis, and the analytical results are shown as the mean ± standard deviation in Table 1. Additionally, the maximum value and minimum value for each curvature of different areas from all taluses were calculated.

**Table 1:** Average curvature from talar radii (‾X ± SD). Note: AM: anteromedial, AL: anterolateral, PL: posterolateral, PM: posteromedial, MP: mid-posterior, MA: mid-anterior

Area	*N*	Curvature(mm)			
		Mean	SD	Min	Max
AM	91	15.97	3.08	10.19	25.51
AL	91	22.06	3.94	14.78	38.74
PL	91	27.95	12.08	16.07	89.98
PM	91	34.17	16.22	14.4	91.41
MP	91	22.44	5.05	15.34	52.98
MA	91	16.97	3.09	10.46	27.87

### Characteristics of talus curvature in different areas

**Fig. 5:**
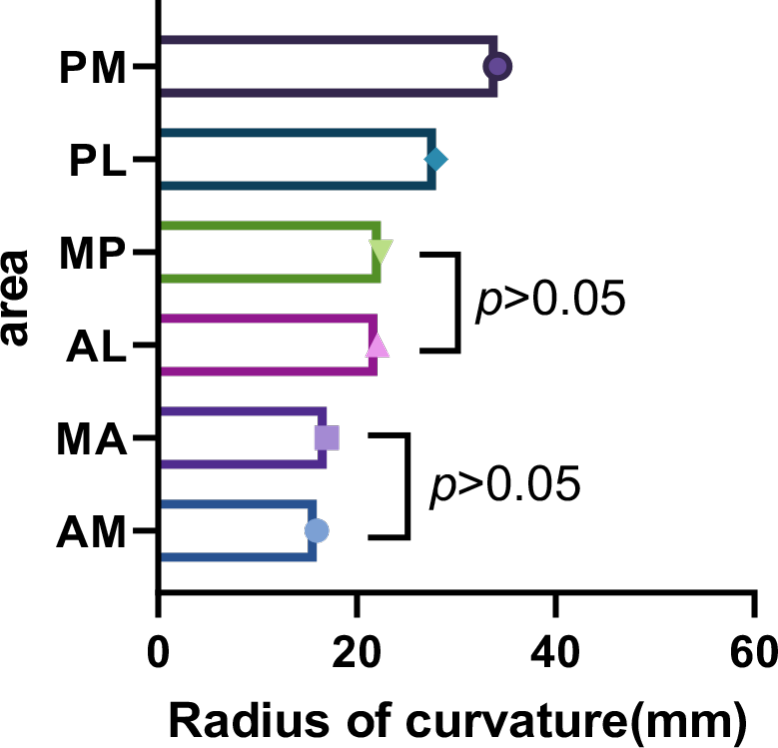
Trends in the size of the talus curvature

After analysis of variance with the LSD method, there were significant differences in talus curvature among the six areas (F = 54.905, *P* = 0.000). Radius of talus curvature from PM is significantly larger than this from PL (*P* = 0.000), radius of talus curvature from PL is significantly larger than this from MP and AL (*P* = 0.000 and *P* = 0.000), radius of talus curvature from AL is significantly larger than this from MA and AM (*P* = 0.000 and *P* = 0.000). The trends in the analytical results were as follows: PM > PL > MP > AL > MA > AM. (see Fig. 5)

### Age-related difference in the curvature of the right talus

The median age was 38 years old, so two groups were categorized. Fifteen talus models in the male population were selected, including 10 specimens less than or equal to 38 years old and 5 specimens older than 38 years. In the statistical results, the average PL radius of specimens older than 38 years old was larger than that of specimens younger than or equal to 38 years old (t=-2.303, *P* = 0.038), and the differences between these two groups in other areas were not significant.

**Fig. 6:**
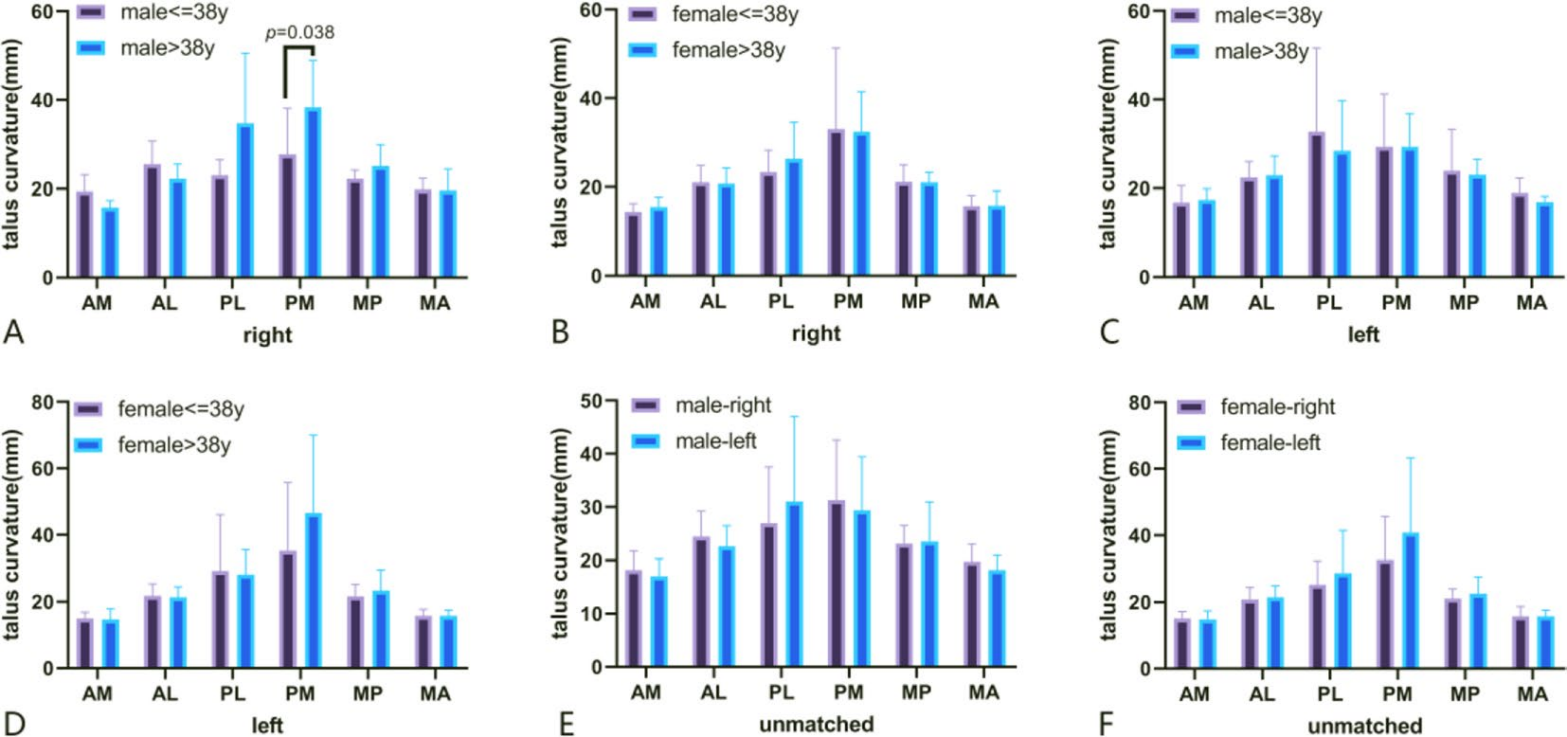
Different talus curvature grouped by age at different areas.(**A**. Comparison of right talus curvature at different ages for males; **B**. Comparison of right talus curvature at different ages for females; **C**. Comparison of left talus curvature at different ages for males; **D**. Comparison of left talus curvature at different ages for females; **E**. Unatched difference between right and left for males; **F**. Unatched difference between right and left for females.)

Twenty-six talar models in the female population were selected, including 10 specimens less than or equal to 38 years old and 16 specimens older than 38 years. The average radius of the six areas in our research between the two groups was not statistically significant(Fig. 6A and B).

### Age-related difference in curvature of the left talus

**Fig. 7:**
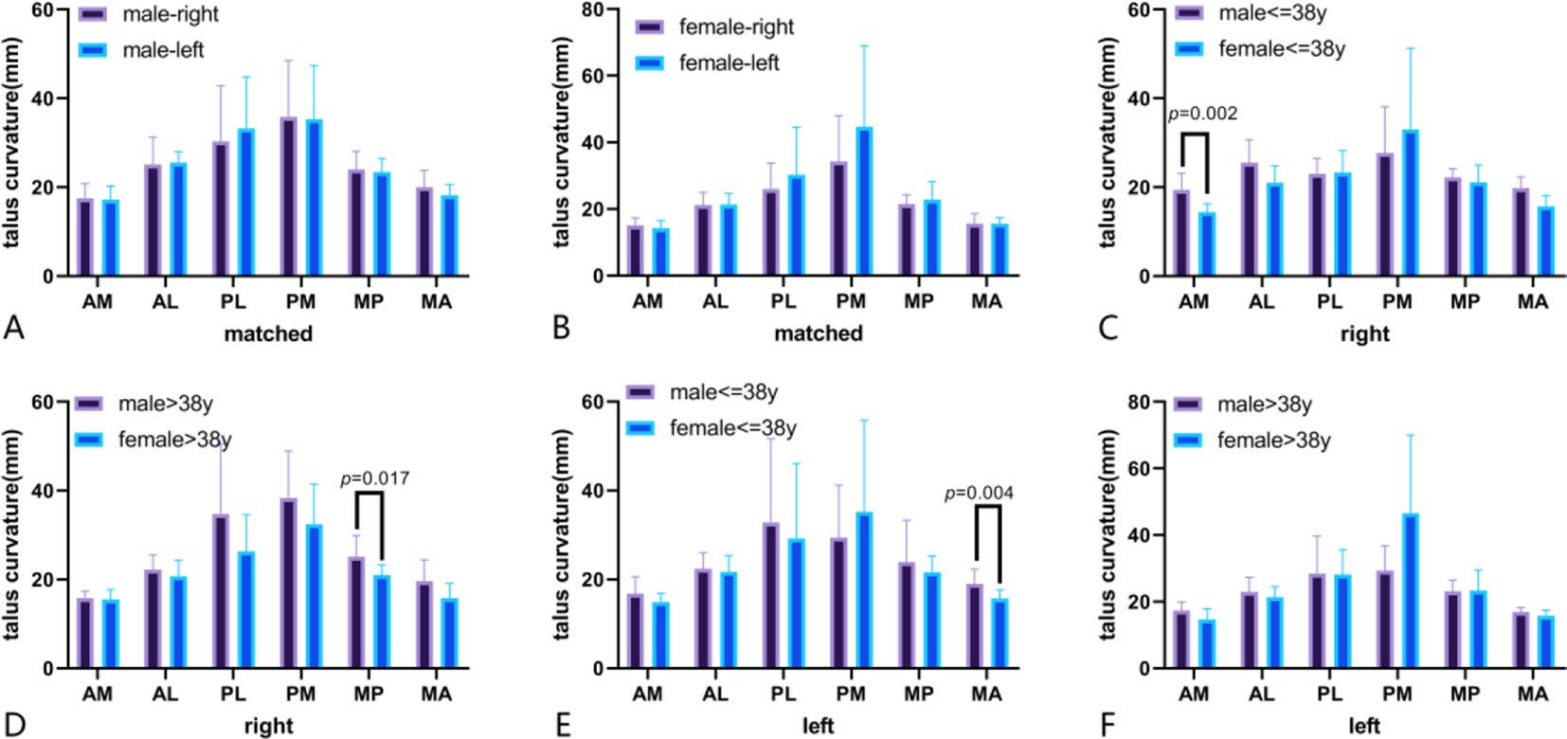
Different talus curvature grouped by sex at different areas.(**A**. Matched difference of talus curvature between right and left for males; **B**. Matched difference of talus curvature between right and left for females; **C**. Comparison of right talus curvature between males and females with age < = 38y; **D**. Comparison of right talus curvature between males and females with age > 38y; **E**. Comparison of left talus curvature between males and females with age < = 38y; **F**. Comparison of left talus curvature between males and females with age > 38y.)

Twenty-two talus models in the male population were selected, including 13 specimens less than or equal to 38 years old and 9 specimens older than 38 years. In the curvature analysis for the six areas, the differences in radius between these two groups were not significant. In the female population, 28 talus models were selected, including 14 specimens less than or equal to 38 years old and 14 specimens older than 38 years. In curvature analysis for six areas, differences in radius in these two groups have no significance(Figs. 6C and 7D).

### Sex-related difference between the left and right talus

Thirty-seven talus models in the male population were included (15 right and 22 left). The statistical results show no significant differences between the right and left sides for the six talus areas. Fifty-four talar models in the female population were reconstructed (26 right and 28 left). The statistical results show no significant differences between the right and left sides for the six talus areas(Fig. 6E and F).

### Matched comparison between the left talus and right talus

Eighteen talus models, including right and left taluses in the male population, were selected, including 9 right taluses and 9 left taluses. After paired *t* test analysis, there were no significant differences between the right and left sides for the six talus areas. Twenty-one bilateral talar models in the female population were selected, and there were no significant differences between the right and left sides for the six talar areas(Fig. 7A and B).

### Differences in the right talus in six areas between males and females

For observers aged < = 38 years, the AM curvature of the right talus in the male group was significantly larger than that in the female group (*P* < 0.01). However, the MP radius in the male group aged > 38 years was larger than that in the female group (*P* < 0.05), and there were no significant differences in other variables between males and females(Fig. 7C and D).

### Differences in the left talus in six areas between males and females

In the age < = 38 years group, the MA curvature of the right talus in males was significantly larger than that in females (*P* < 0.01), and there were no significant differences in other variables between males and females(Fig. 7E and F).

### Curvature difference between males and females

**Fig. 8:**
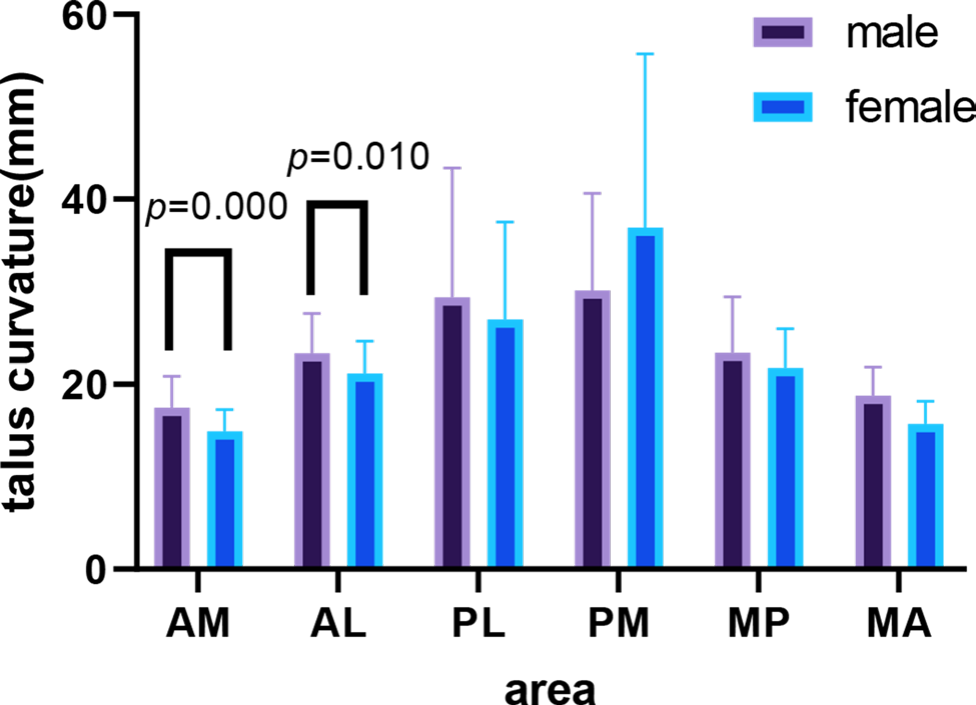
Differences between males and females in six areas

The curvature of the AM for males was significantly larger than that for females (t = 4.25, *P* = 0.000), and the curvature of the AL for males was larger than that for females (t = 2.629, *P* = 0.010); however, there were no significant differences for all radii of the talus at the PL/PM/MP/MA between males and females (Fig. 8).

### Fitting radius of talus curvature

The average fitted curvature for the talus was 20.52 ± 1.95 mm (range 15.18–25.30). The fitting radius of the talus for males (21.90 ± 1.97 mm) was significantly greater than that for females (19.57 ± 1.26 mm) (t = 6.894, *P* = 000). The fitting radius of the talus on the right side (20.69 ± 2.00 mm) was similar to that on the left side (20.37 ± 1.92 mm) (t = 0.783, *P* = 0.436).

### Correlation analysis between age and fitting talus curvature radius

There was no correlation between age and talus curvature in the general analysis (*r*=-0.015, *P* = 0.886). Stratified by sex, the fitting radius for the male and female populations was not related to age (*r* = 0.073, *P* = 0.666; *r* = 0.157, *P* = 0.258); stratified by left and right, the fitting radius for the right and left talus was not related to age (*r*=-0.061, *P* = 0.704; *r* = 0.014, *P* = 0.921).

## Discussion

Talus ankle replacement is the ultimate treatment for severe ankle diseases. At present, there have been many studies on ankle joint replacement and prosthesis design because the characteristic shape of the human talus vault is irregular and asymmetric, which urges the human ankle joint to complete complex plantar flexion and dorsiflexion. However, it was found that the symmetrical design of the talar prosthesis at present could not meet the patient’s activities of flexing the metatarsal dorsum and stretching. Therefore, researchers actively understand the anatomical characteristics of the talus dome and, based on this, design prosthesis components close to the talus structure.

Conversely, a study suggested that TAR should be avoided for severe talar osteonecrosis because of subsidence of the talar component caused by poor bone quality [18]. The most complicated was reported as subsidence from the talus component, and the incidence varies greatly (1–15%) [19, 20]. Complications were observed that predispose the structure to fragility and subsidence under the talar component after total ankle arthroplasty (TAA) caused by misposition [21]. The loosening rate of the talus component after TAR has reached 92% [22], and the salvage treatment is ankle arthrodesis, which increases the loss of ankle motion. Dorsiflexing of the talar prosthesis during TAA (INFINITY ankle system) was possibly associated with talar component subsidence and limited dorsiflexion range [23]. We supposed that complications are partly related to the design of the talus component.

In a talar topography study of CT images of 21 Caucasian U.S. adult patients suffered from ankle osteoarthritis [24], and the radius of the talar dome was 20.7 ± 2.6 mm. This result was similar to our measurement; however, our result further disclosed that the curvature between males and females was different in the two different measurement methods, and the radius in males was larger than that in females. In addition, the curvature at AM and AL in the male group was larger than that in the female group. For right talus in male group, radius of AM with age < = 38years and MP with age > = 38years were greater than in female; for left talus in male group, radius of AM with age < = 38years was greater than in female.

The shape of the talus should be constant when it grows; however, a study reported that age is significantly negatively related to curvature at the superior trochlea [25]. In this study, stratified analysis by sex between the age < = 38 years group and the age > 38 years group in the left and right sides showed no significant difference during this research; moreover, there was no significant relation between age and fitting talus curvature radius.

**Fig. 9:**
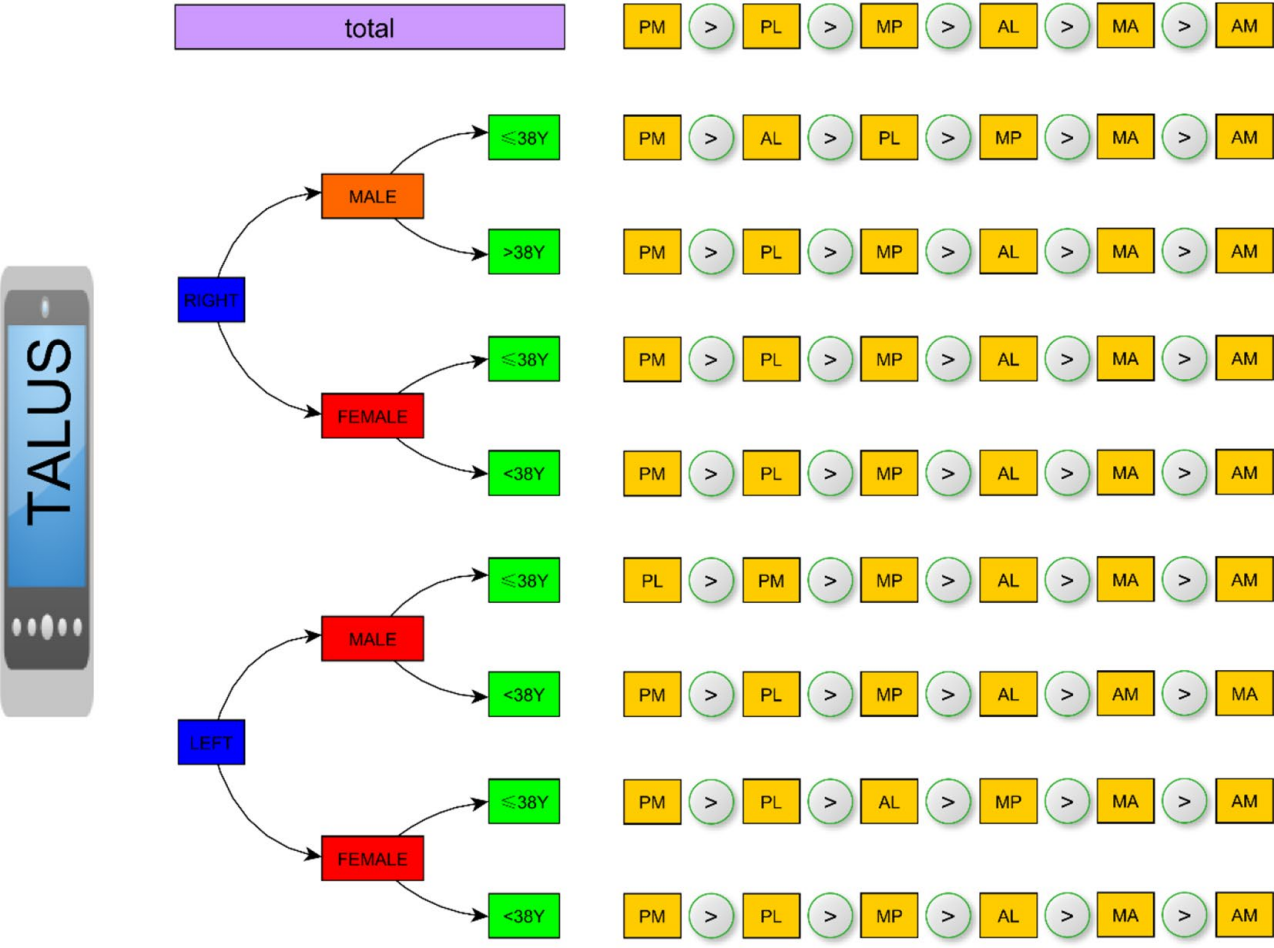
Change trend for different subject populations in six areas

A novel treatment of customized total talar prosthesis in combination with the tibial component of TAA for ankle revision has been verified to improve JSSF ankle hindfoot scale scores, subjective pain, and range of motion in the ankle [26, 27]. Increasing the surface for talus coverage will help stabilize the talus component in TAR [28], which helps surgeons decide the size of the talus. It was found [29] that medial crests of the talar dome consisted of two circles with different radii, of which the posterior radius was larger, and the radius of the anterior lateral crests was nearly the same as that of the posterior lateral crests. A Chinese cohort study including 71 talus models also showed that the mean radii of lateral-anterior, lateral-posterior, medial-anterior, and medial-posterior were 19.23 ± 2.47 mm, 18.76 ± 2.90 mm, 17.02 ± 3.49 mm, and 22.75 ± 3.04 mm, respectively; however, these results did not consider sex and age [29]. This may be the reason for the difference in our research results. Our results revealed the curvature difference of the uppersurface from the talus, which was summarized as follows: PM > PL > MP > AL > MA > AM. However, slight differences were identified when confounding factors were considered, such as age and sex (Fig. 9). Therefore, we strongly recommend that joint surgeons consider these factors before TAR.

Of course, our study has some limitations, including (1) subjects from Northren China, which cannot represent all Chinese people; (2) the size of the sample is small, and we need more samples to explore the talus characteristics; and (3) biomechanical analysis by FEA technology on the talus needs to be executed to optimize the performance of the talus component.

## Conclusions

The talus trochlea with different radii allow the ankle joint to complete complex rotation, flexion, and extension activities during walking. Excellent design of the talus component is likely associated with fewer complications after TAR. To our knowledge, this is the first study on the characteristics of talus from Chinese people of different ages and sexes to reduce probable confounding factors. There was no significant relationship between age and talus curvature for either males or females. The average radius of the talus in the male population was larger than that in the female population. The radius of curvature in the posterior area was significantly larger than that in the anterior area. We recommend that this characteristic of the talus trochlea should be considered when designing the talus component in TAR. Our manuscript supplies very important anatomical evidence for designing talus components with different radii curvatures.

### Electronic supplementary material

Below is the link to the electronic supplementary material.

Supplementary Material 1

## Data Availability

No datasets were generated or analysed during the current study.

## Abbreviations

AM: Anteromedial
PM: Posteromedial
AL: Anterolateral
PL: Posterolateral
MA: Mid-anterior
MP: Mid-posterior
TAR: Total ankle replacement
FEA: Finite element analysis
OAT: Osteochondral autologous transplantation
3D: Three‑dimensional
TTR: Total talus replacement
BMI: Body mass index
ROI: Region of interest
OCS: Object coordinate system
WCS: World coordinate system
TAA: Total ankle arthroplasty
KBD: Kashin-Beck disease
