# Supplementary material for: Different radius of curvature at the talus trochlea from northern Chinese population measured using 3D model
Source: J Orthop Surg Res. 2024 Apr 27;19:266. doi: 10.1186/s13018-024-04751-7 (PMC11055296; doi:10.1186/s13018-024-04751-7)
Supplement: Supplementary file 1 — Supplementary Material 1 [file 13018_2024_4751_MOESM1_ESM.docx]

(Fig.6)**Table 2**. Comparison of talus curvature from males at different ages for six areas (‾X±SD). Note: AM: anteromedial, AL: anterolateral, PL: posterolateral, PM: posteromedial, MP: mid-posterior, MA: mid-anterior. Bold means *P* <0.05

| **Right** | **Male** | | **t/*p*** | **Female** | | **t/*p*** |
| --- | --- | --- | --- | --- | --- | --- |
|  | <=38y(10) | >38y(5) |  | <=38y(10) | >38y(16) |  |
| **AM(mm)** | 19.36±3.78 | 15.80±1.56 | 1.986/0.069 | 14.41±1.87 | 15.47±2.24 | -1.244/0.226 |
| **AL(mm)** | 25.53±5.18 | 22.19±3.34 | 1.299/0.216 | 21.03±3.79 | 20.72±3.55 | 0.208/0.837 |
| **PL(mm)** | 23.03±3.49 | 34.71±15.85 | -2.303/**0.038** | 23.32±4.94 | 26.32±8.24 | -1.036/0.311 |
| **PM(mm)** | 27.71±10.43 | 38.31±10.64 | -1.844/0.088 | 33.04±18.27 | 32.45±9.02 | 0.110/-0.913 |
| **MP(mm)** | 22.22±1.97 | 25.06±4.86 | -1.643/0.124 | 21.07±3.89 | 21.00±2.29 | 0.055/0.956 |
| **MA(mm)** | 19.80±2.54 | 19.62±4.82 | 0.095/0.925 | 15.65±2.48 | 15.75±3.40 | -0.078/0.938 |

(Fig. 7).**Table 3**. Comparison of left talus curvature from males at different ages for different areas (‾X±SD). Note: AM: anteromedial, AL: anterolateral, PL: posterolateral, PM: posteromedial, MP: mid-posterior, MA: mid-anterior.

| **Left** | **Male** | | **t/*p*** | **Female** | | **t/*p*** |
| --- | --- | --- | --- | --- | --- | --- |
|  | <=38y(13) | >38y(9) |  | <=38y(14) | >38y(14) |  |
| **AM(mm)** | 16.79±3.77 | 17.36±2.52 | -0.389/0.701 | 14.89±1.95 | 14.72±3.14 | 0.175/0.862 |
| **AL(mm)** | 22.39±3.62 | 22.89±4.39 | -0.291/0.774 | 21.68±3.71 | 21.36±3.11 | 0.244/0.809 |
| **PL(mm)** | 32.77±18.86 | 28.45±11.26 | 0.613/0.547 | 29.18±16.91 | 28.18±7.48 | 0.203/0.841 |
| **PM(mm)** | 29.37±11.84 | 29.35±7.44 | 0.005/0.96 | 35.24±20.54 | 46.57±23.46 | -1.359/0.186 |
| **MP(mm)** | 23.95±9.31 | 23.02±3.51 | 0.288/0.776 | 21.61±3.62 | 23.36±6.20 | -0.912/0.37 |
| **MA(mm)** | 19.01±3.25 | 16.87±1.33 | 1.855/0.078 | 15.72±1.95 | 15.79±1.73 | -0.092/0.927 |

(Fig.8).**Table 4**. Difference between right and left for males and females (‾X±SD). Note: AM: anteromedial, AL: anterolateral, PL: posterolateral, PM: posteromedial, MP: mid-posterior, MA: mid-anterior.

| **Unmatched** | **Male** | | **t/*p*** | **Female** | | **t/*p*** |
| --- | --- | --- | --- | --- | --- | --- |
|  | Right(15) | Left(22) |  | Right(26) | Left(28) |  |
| **AM(mm)** | 18.17±3.59 | 17.02±3.26 | 1.009/0.320 | 15.06±2.13 | 14.81±2.57 | 0.400/0.690 |
| **AL(mm)** | 24.42±4.80 | 22.60±3.86 | 1.276/0.210 | 20.84±3.57 | 21.52±3.37 | -0.718/0.476 |
| **PL(mm)** | 26.92±10.59 | 31.00±16.00 | -0.864/0.393 | 25.17±7.19 | 28.68±12.84 | -1.227/0.225 |
| **PM(mm)** | 31.25±11.35 | 29.36±10.06 | 0.531/0.599 | 32.67±13.00 | 40.91±22.39 | -1.636/0.108 |
| **MP(mm)** | 23.16±3.34 | 23.57±7.38 | -0.198/0.844 | 21.03±2.93 | 22.48±5.06 | -1.275/0.208 |
| **MA(mm)** | 19.74±3.28 | 18.13±2.80 | 1.597/0.119 | 15.71±3.02 | 15.76±1.81 | -0.063/0.950 |

(Fig.9) **Table 5**. Matched difference between right and left for males and females (‾X±SD). Note: AM: anteromedial, AL: anterolateral, PL: posterolateral, PM: posteromedial, MP: mid-posterior, MA: mid-anterior.

| **Matched** | **Male** | | **t/*p*** | **Female** | | **t/*p*** |
| --- | --- | --- | --- | --- | --- | --- |
|  | Right(9) | Left(9) |  | Right(21) | Left(21) |  |
| **AM(mm)** | 17.48±3.32 | 17.16±3.11 | 0.383/0.711 | 15.05±2.24 | 14.27±2.26 | 1.255/0.224 |
| **AL(mm)** | 25.07±6.26 | 25.48±2.49 | -0.230/0.824 | 21.14±3.82 | 21.29±3.39 | -0.255/0.801 |
| **PL(mm)** | 30.33±12.55 | 33.26±11.62 | -0.757/0.471 | 25.98±7.78 | 30.30±14.28 | -1.348/0.193 |
| **PM(mm)** | 35.82±12.71 | 35.37±12.06 | 0.125/0.904 | 34.20±13.80 | 44.70±24.35 | -1.782/0.090 |
| **MP(mm)** | 24.01±4.12 | 23.41±3.10 | 0.357/0.731 | 21.48±2.78 | 22.85±5.38 | -1.231/0.232 |
| **MA(mm)** | 19.97±3.79 | 18.16±2.45 | 1.349/0.214 | 15.59±3.03 | 15.64±1.74 | -0.097/0.924 |

(Fig.11) **Table 6**. Different right talus radii between males and females in the two age groups (‾X±SD). Note: AM: anteromedial, AL: anterolateral, PL: posterolateral, PM: posteromedial, MP: mid-posterior, MA: mid-anterior. Bold means *P*<0.05.

| **Right** | **<=38y** | | **t/*p*** | **>38y** | | **t/*p*** |
| --- | --- | --- | --- | --- | --- | --- |
|  | Male(10) | Female(10) |  | Male(5) | Female(16) |  |
| **AM(mm)** | 19.36±3.78 | 14.41±1.87 | 3.703/**0.002** | 15.80±1.56 | 15.47±2.24 | 0.309/0.761 |
| **AL(mm)** | 25.53±5.18 | 21.03±3.79 | 2.217/0.04 | 22.19±3.34 | 20.72±3.55 | 0.817/0.424 |
| **PL(mm)** | 23.03±3.49 | 23.32±4.94 | -0.152/0.881 | 34.71±15.85 | 26.32±8.24 | 1.587/0.129 |
| **PM(mm)** | 27.71±10.43 | 33.04±18.27 | -0.800/0.434 | 38.31±10.64 | 32.45±9.02 | 1.219/0.238 |
| **MP(mm)** | 22.22±1.97 | 21.07±3.89 | 0.830/0.417 | 25.06±4.86 | 21.00±2.29 | 2.619/**0.017** |
| **MA(mm)** | 19.80±2.54 | 15.65±2.48 | 3.694/0.002 | 19.62±4.82 | 15.75±3.40 | 2.019/0.058 |

(Fig.12) **Table 7**. Different left talar radii between males and females in the two age groups (‾X±SD). Note: AM: anteromedial, AL: anterolateral, PL: posterolateral, PM: posteromedial, MP: mid-posterior, MA: mid-anterior. Bold means *P*<0.01.

| **left** | **<=38y** | | **t/*p*** | **>38y** | | **t/*p*** |
| --- | --- | --- | --- | --- | --- | --- |
|  | male(13) | female(14) |  | male(9) | female(14) |  |
| **AM(mm)** | 16.79±3.77 | 14.89±1.95 | 1.667/0.108 | 17.36±2.52 | 14.72±3.14 | 2.111/0.047 |
| **AL(mm)** | 22.39±3.62 | 21.68±3.71 | 0.506/0.617 | 22.89±4.39 | 21.36±3.11 | 0.980/0.338 |
| **PL(mm)** | 32.77±18.86 | 29.18±16.91 | 0.521/0.607 | 28.45±11.26 | 28.18±7.48 | 0.070/0.945 |
| **PM(mm)** | 29.37±11.84 | 35.24±20.54 | -0.901/0.376 | 29.35±7.44 | 46.57±23.46 | -2.120/0.046 |
| **MP(mm)** | 23.95±9.31 | 21.61±3.62 | 0.876/0.389 | 23.01±3.51 | 23.36±6.20 | -0.151/0.882 |
| **MA(mm)** | 19.01±3.25 | 15.72±1.95 | 3.211/**0.004** | 16.87±1.33 | 15.79±1.73 | 1.601/0.124 |
